# Toxicology evaluation of overdose hydroxychloroquine on zebrafish (*Danio rerio*) embryos

**DOI:** 10.1038/s41598-022-23187-9

**Published:** 2022-10-29

**Authors:** Min Luo, Dan Xie, Ziyuan Lin, Huaqin Sun, Yanyan Liu

**Affiliations:** 1 Prenatal Diagnosis Center, Department of Obstetrics & Gynecologic, Key Laboratory of Birth Defects and Related Diseases of Women and Children (Sichuan University), Ministry of Education, West China Second University Hospital, Sichuan University, Chengdu, 610041 People’s Republic of China; 2 SCU-CUHK Joint Laboratory for Reproductive Medicine, Key Laboratory of Birth Defects and Related Diseases of Women and Children (Sichuan University), Ministry of Education, Department of Pediatrics, West China Second University Hospital, Sichuan University, Chengdu, 610041 People’s Republic of China

**Keywords:** Disease model, Drug safety, Toxicology, Risk factors

## Abstract

Potential risks of treatment with hydroxychloroquine (HCQ) include QT interval prolongation, hypoglycemia, a wide range of neuropsychiatric manifestations, hematotoxicity, and potential genetic defects. HCQ is extremely toxic when used in overdose and can lead to tachycardia, hypotension, known central nervous system, transmission defects, hypokalemia and other manifestations in individuals. The mechanism of excessive HCQ leading to these manifestations is still unclear. In this paper, overdose HCQ at different concentrations was used to treat zebrafish embryos, and the phenomena like human beings were obtained, such as increased heart rate and nervous system inhibition. With the increase of concentration to 100 μM, embryo mortality and malformation rate increased and hatching rate decreased, in situ hybridization showed abnormal differentiation of embryo germ layers and formation of vital organs. We selected embryos treated with 50 μM HCQ, in which concentration the mortality rate, hatching rate and malformation rate of the embryos were like those of the control group, for transcriptome analysis. Although the above indexes did not change significantly, the molecular changes related to the development of the heart, eye, nerve and other important organs were significant. This study provides useful information for further research on the toxicity mechanism of HCQ overdose, and provides some insight that can guide future studies in humans.

## Introduction

Hydroxychloroquine (HCQ) is a 4-aminoquinoline derivative prepared by beta hydroxylation of chloroquine. It is used in the treatment of malaria, rheumatoid arthritis, and systemic lupus erythematosus (SLE)^1^. Over the last several years, there has been heightened interest in HCQ because of it being a candidate drug for the treatment and/or prophylaxis of coronavirus disease 2019 (COVID-19). Between March 30, 2020, and June 15, 2020, HCQ was granted emergency use authorization by the Food and Drug Administration, allowing it to be used for COVID-19 outside the clinical trial setting, resulting in widespread use during that time window^2,3^. Later investigations demonstrated increased morbidity and mortality, largely owing to cardiotoxicity associated with HCQ. The FDA retracted its EUA on June 15, 2020, warning not to use these agents to treat COVID-19^4,5^.

HCQ is generally well tolerated, but clinicians and patients should be aware of serious adverse events that can occur, even during short courses of treatment. Potential risks of treatment include QT interval prolongation (a measurement made on an electrocardiogram, calculated as the time from the start of the Q wave to the end of the T wave), hypoglycemia, neuropsychiatric effects, drug–drug interactions and idiosyncratic hypersensitivity reactions^6^. Genetic variability in metabolism of these drugs is considerable and influences their safety and effectiveness^7^. HCQ overdoses are rare, and very serious. Life-threatening symptoms may occur within 30 min with very rapid progression to death within a few hours^1^. A previous study found that a 16-year-old girl ingested a handful of HCQ 200 mg, 30 min before presentation and presented with tachycardia (heart rate 110 beats/min), hypotension (systolic blood pressure 63 mmHg), central nervous system depression, conduction defects (QRS = 0.14 ms), and hypokalemia (K = 2.1 meq/L)^1^. Awareness of the onset of symptoms, potential severity, toxidrome, and treatment recommendations will greatly aid in patient survival. Unfortunately, treatment recommendations for this drug overdose are not well established and are often unconventional.

The study of the effect of excessive HCQ on the heart, nerves and other important organs and the mechanism of toxicity will provide a theoretical basis for the treatment of excessive HCQ intake. In this study, zebrafish was used as a model organism to study the toxic effects of excessive HCQ on the body. We believe that our study provides insights into the toxicity of HCQ in the animal model studied. Furthermore, considering that zebrafish is considered a good translational model for humans^8^, this study provides some insight that can guide future studies in humans.

## Results

### HCQ-induced developmental defects

#### Mortality

Zebrafish embryo mortality was recorded at 8 h postfertilization (hpf), 1 day postfertilization (dpf), 2 dpf, 3 dpf, and 4 dpf under the influence of 0 μM, 12.5 μM, 25 μM, 50 μM, 100 μM, and 200 μM HCQ that was administered at 0 hpf.

**Figure 1:**
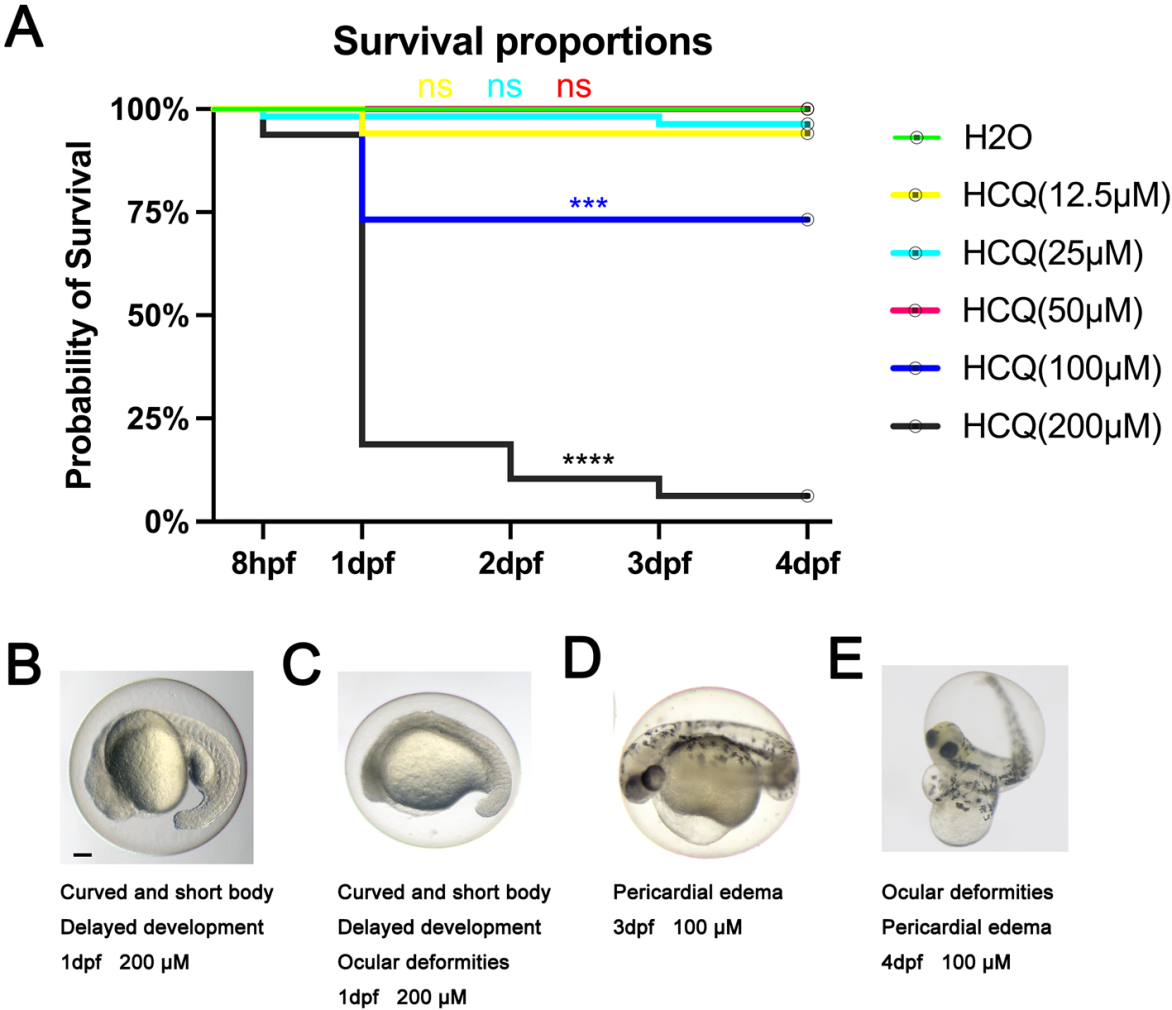
Survival curves of zebrafish embryos exposed to different concentrations of HCQ from 8 hpf to 4 dpf. Lines of different colors represent different groups, and green represents the control group. (**A**) Unpaired t test was used to test for statistical significance *P < 0.05; **P < 0.01; ***P < 0.001; ****P < 0.0001, the same as below. (**B**–**E**) Images are examples of developmental malformations. Scale bars, 100 μM.

The cumulative mortality of the control and experimental groups at concentrations of HCQ not exceeding 50 μM remained low (p > 0.05) as follows: 0 μM (0.0%), 12.5 μM (5.9%), 25 μM (3.6%), and 50 μM (0.0%). Prior to completion of 1 dpf, the experimental group mortality rate at concentrations exceeding 50 μM remained low. On reaching 1 dpf however, the cumulative mortality rate at 100 μM increased significantly to 26.8% (p < 0.05). The mortality rate at 200 μM further surged significantly to 81.2% at 1 dpf, 89.6% at 2 dpf, and reached a maximum of 93.8% at 3 dpf (p < 0.05) (Fig. 1A).

#### Deformity

While embryos in the control group were found to develop normally during all exposure periods. the HCQ group developed abnormalities at concentrations higher than 50 μM. As shown in Fig. 1B–E, these included tail deformities, scoliosis, yolk deformities, delayed development, pericardial edema, and ocular deformities, with the latter two being the most obvious. Similar to effect observed in terms of embryo mortality, the severity of abnormalities including pericardial edema, yolk sac deformities, and ocular deformities increased with increasing HCQ concentrations. Embryos exposed to 200 μM HCQ exhibited the severest deformities.

#### Hatching rate

**Figure 2:**
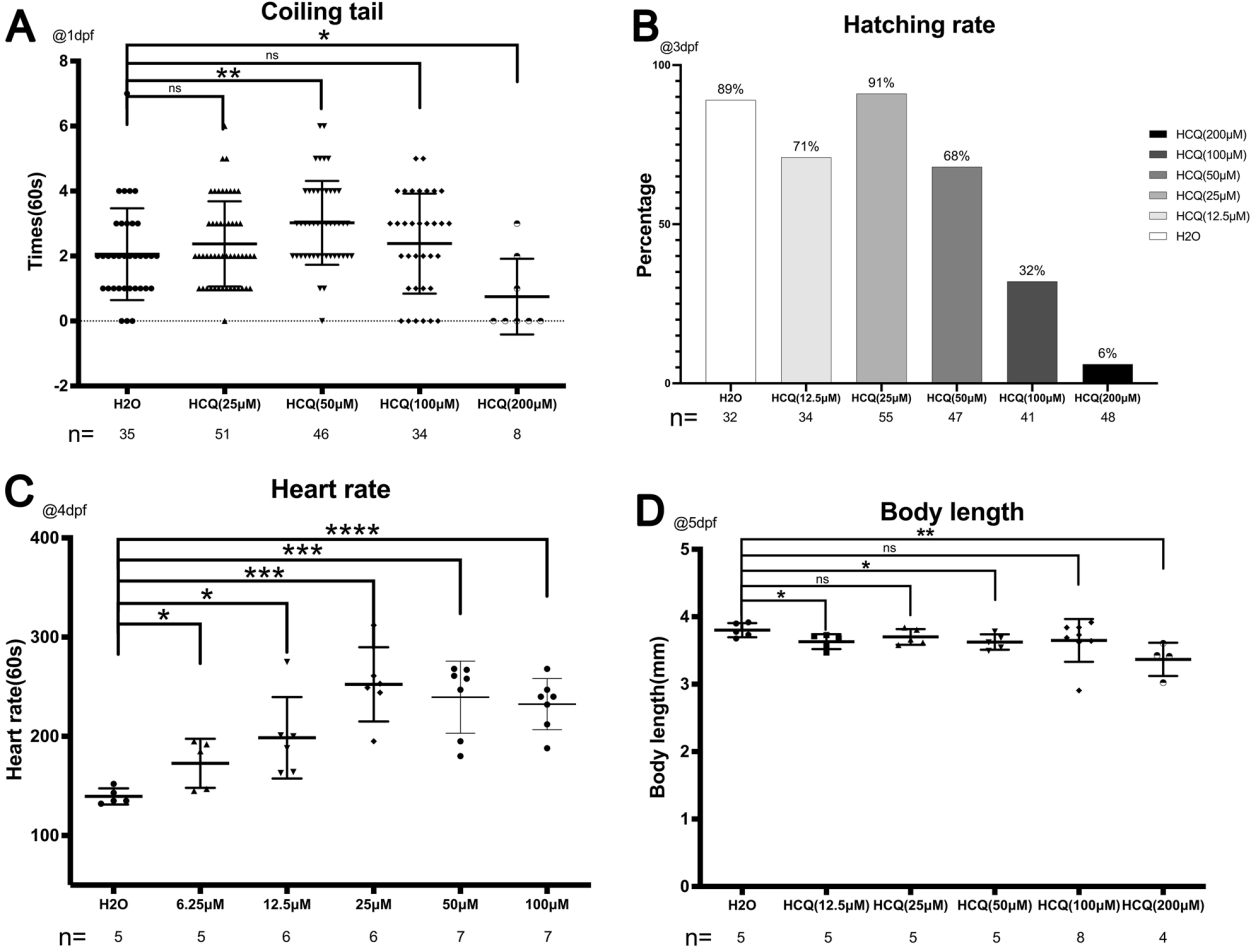
(**A**) Scatter plot and statistical results of the number of coiling tail at 1 dpf. (**B**) Scatter plot and statistical results of hatching rate in 3 dpf. (**C**) Scatterplot and statistical results of heart rate in 4 dpf. (**D**) Scatter plot and statistical results of body length in 5 dpf.

At 3 dpf, the hatching rate of the control group was 89%. At HCQ concentrations below 50 μM, the hatching rate was 71% (12.5 μM), 91% (25 μM), and 68% (50 μM). At concentrations above 50 μM, the hatching rate decreased rapidly to 32% (100 μM) and 6% (200 μM) (Fig. 2B), which may be related to the increase in embryo mortality (26.8% at 100 μM, and 93.8% at 200 μM) (Fig. 1A) at similar HCQ concentrations. At 96 hpf, all the live embryos were found to be dechorionated (Fig. 2B).

#### Heart rate

The heart rates of the zebrafish embryos were recorded at 4 dpf. Towards this end, heart rates of selected embryos from each group were recorded for 20 s post stabilization of the embryonic heartbeat at room temperature for 10 min^9^.

The heart rate of the control group was 139.4 ± 8.1 beats per minute, which was increased to 172.8 ± 24.7 (6.25 μM), 198.5 ± 41.0 (12.5 μM), 252.3 ± 37.4 (25 μM), 239.4 ± 36.4 (50 μM), and 232.4 ± 25.8 (100 μM) bpm post exposure to HCQ, thereby demonstrating that HCQ affects cardiac contractility of zebrafish embryos.

These results indicate that HCQ significantly increases the heart rate of zebrafish embryos (p < 0.05), in a dose-dependent manner. However, the effect was slower at concentrations above 25 μM, which may be a consequence of the cardiotoxic potential of high HCQ concentrations^10,11^ (Fig. 2C).

#### Body length

The body length of zebrafish was found to decrease with increasing HCQ concentrations at 5 dpf. While the body length of zebrafish in the control group was 3.8 ± 0.1 mm, that of the experimental group was 3.6 ± 0.1 mm (12.5 μM), 3.7 ± 0.1 mm (25 μM), 3.6 ± 0.1 mm (50 μM), 3.6 ± 0.3 mm (100 μM), and 3.3 ± 0.2 mm (200 μM). Further, the experimental groups exposed to 12.5 μM, 50 μM and 200 μM HCQ differed significantly from the control group, with the most obvious decrease in body length observed at 200 μM (Fig. 2D).

### Behavior

Spontaneous embryonic movements in the yolk sac were recorded for 1 min at 1 dpf (Fig. 2A). The number of tail coilings per minute in the control group was 2.1 ± 1.4 which was significantly different from that observed at 25 μM (2.4 ± 1.3 times/min) and 100 μM (2.4 ± 1.5 times/min). While the 50 μM group (3.0 ± 1.3 times/min) showed a significant increase (p < 0.005), the 200 μM group (0.8 ± 1.2 times/min) demonstrated a significant decrease (p < 0.05).

As evident from Supplementary video 1, the movements of larvae in terms of swimming distance and velocity in the HCQ group showed reduced responsiveness to external stimuli as compared to that in the control group at 3 dpf. This effect was increasingly apparent with increasing concentrations of HCQ.

Furthermore, replacing the 50 μM HCQ group experimental solution with the same egg water as the control group at 3 dpf, we observed a recovery of responsiveness to external stimuli at 4pdf (24 h later). Therefore, it may be inferred that HCQ interferes, although reversibly with spontaneous zebrafish movements.

### In situ hybridization

To determine the effect of HCQ on zebrafish embryonic tissue development, the control as well as the 50 μM and 100 μM HCQ-treated groups were continually monitored.

**Figure 3:**
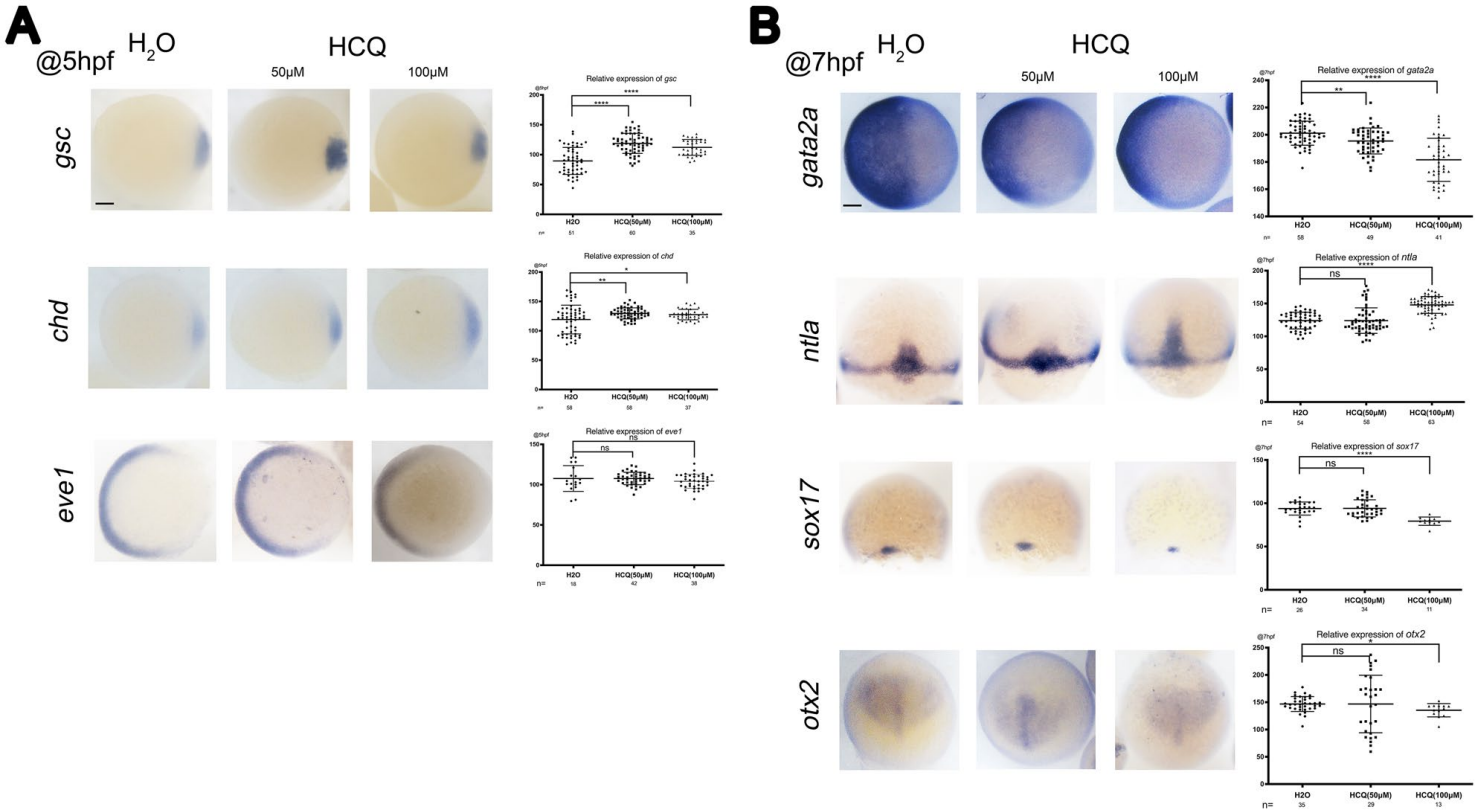
Germ layer marker expression detected by ISH during gastrulation at 5 hpf (**A**) and 7 hpf (**B**). Orientation: *ntla* and *sox17*, dorsal views with animal pole to the top at 75% epiboly stage; dorsal-animal views with anterior towards the top: *otx2*; others, animal-pole view (*eve1*, *chd*, *gsc* and *gata2a*) with dorsal to the right. The scatter plot on the right corresponds to the statistical results of hybridization signals. Scale bars, 100 μM.

At 5 hpf, whole-mount in situ hybridization (WISH) revealed significant increase in the dorsal organizer markers *gsc* and *chd* in embryos exposed to 50 μM and100 μM HCQ at the shield stage (p < 0.05). In these groups HCQ changed the fate of dorsal–ventral cells and expanded the dorsal area, thus indicating that it can developmentally regulate the dorsoventral axis in zebrafish. In contrast, the expression of the ventral marker *eve1* did not differ significantly between the 50 μM (p > 0.05), 100 μM (p > 0.05) and control groups (Fig. 3A).

The expression of the epidermal marker *gata2a* was decreased throughout the ectoderm in the shielding stage (7 hpf stage) in the 50 μM (p < 0.05) and 100 μM (p < 0.05) groups, and zebrafish in these groups exhibited significantly reduced ectodermal development (Fig. 3B).

Additionally, similar to the observation made for marker genes at 7 hpf, the expression level of the mesoderm marker *ntla* was not significantly different in the 50 μM group (p > 0.05), but increased significantly in the 100 μM group (p < 0.05) as compared to that of the control group. Therefore, 100 μM HCQ upregulates development of zebrafish mesoderm (Fig. 3B).

The endoderm marker *sox17* and anterior neuroectoderm marker *otx2* did not differ significantly between the 50 μM and control groups (p > 0.05), but were significantly reduced in the 100 μM group (p < 0.05). Therefore, 100 μM HCQ downregulates the development of zebrafish endoderm (intestines, liver, and other internal organs) and anterior neuroectoderm (Fig. 3B).

**Figure 4:**
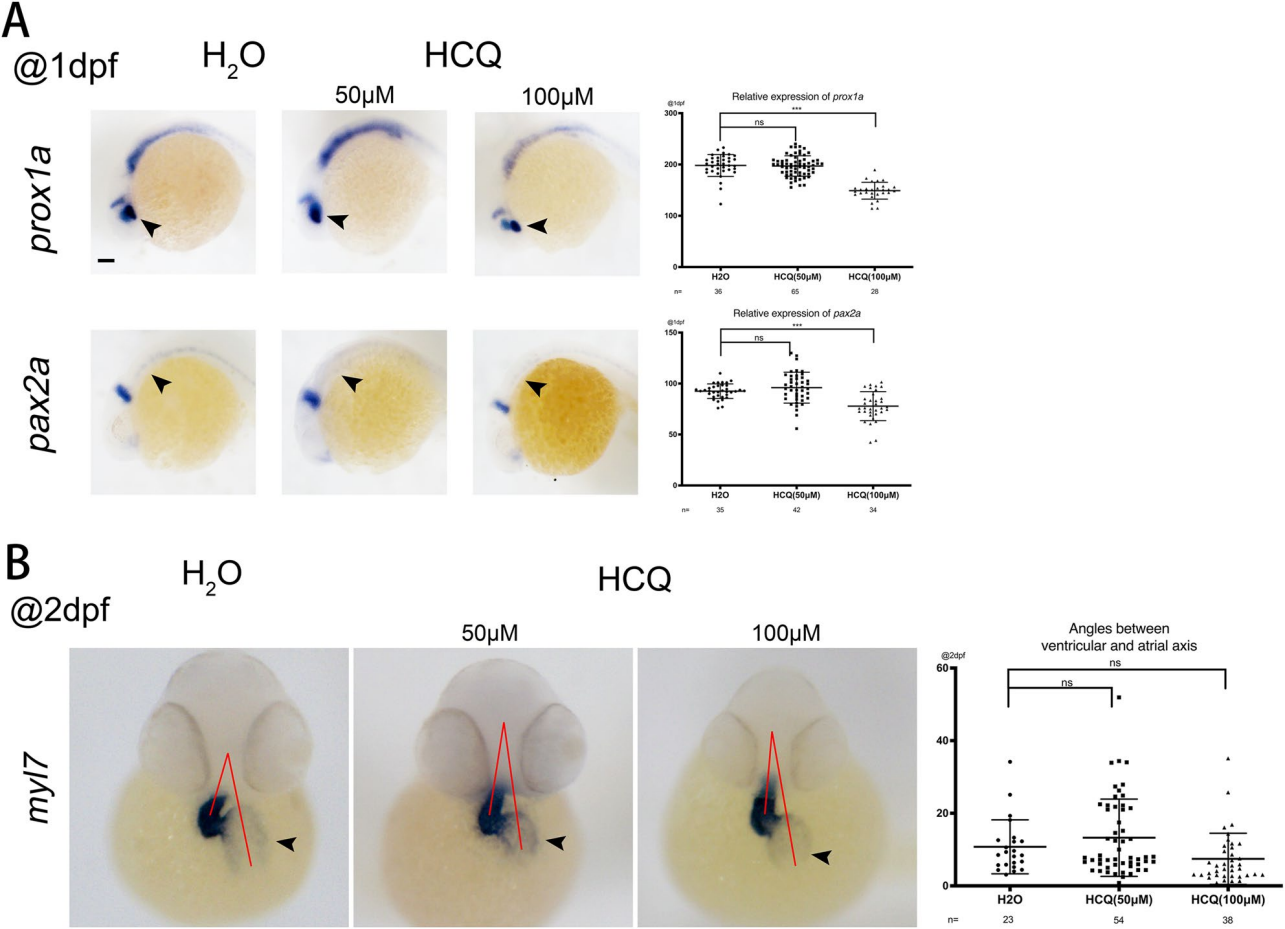
(**A**) Marker expression at 24 hpf. Lateral views with anterior to the left. (**B**) Heart morphology (marked by the pan-cardiomyocyte marker *myl7*) at 48 hpf. Embryo orientation: ventral view with the anterior at the top. Arrows point to the atrium; red lines demonstrate the acute angle formed between the atrial and ventricular axes. The scatter plot on the right corresponds to the statistical results of angles.

*Prox1a* is a target of β-catenin-TCF/LEF signaling in the zebrafish eye^12^, and *Pax2a* is expressed in several structures, including the immature eye, mesoderm, nervous system, neural keel, and pronephros. At 1 dpf, the expression levels of *prox1a* and *pax2a* were not significantly different between the 50 μM and control groups (p > 0.05), but were significantly lowered in the 100 μM group (p < 0.05). A decrease in the expression of these two markers indicates that HCQ downregulates development of the zebrafish eye, nervous system, and renal system (Fig. 4A).

*Myl7*, a cardiac marker is expressed in cardiomyocytes throughout the entire heart. At 2 dpf, the expression of *myl7* was not significantly different between the control, 50 μM (p > 0.05), and 100 μM (p > 0.05) groups (Fig. 4B).

### Transcriptome analysis

The molecular mechanism of HCQ treatment in terms of its effect on zebrafish embryonic RNA levels was analyzed by using a transcriptome assay (NCBI SRA accession: PRJNA879284).

**Figure 5:**
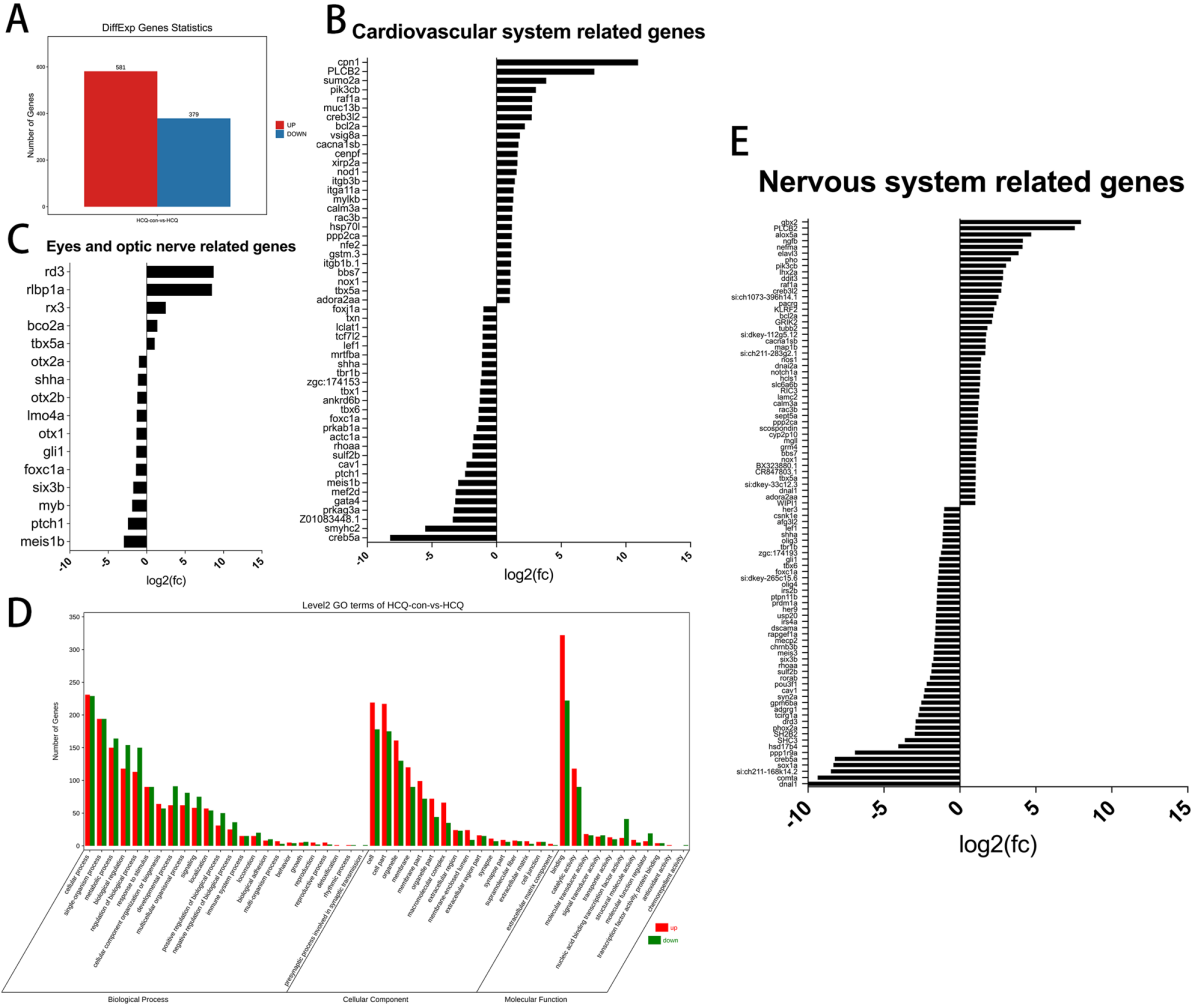
(**A**) Overall statistics of differential genes. (**B**) Significantly differential genes associated with the development of the cardiovascular system. (**C**) Significantly differential genes associated with eye and optic nerve development. (**D**) Number of significantly differentially expressed genes for biological processes, cellular components, and molecular functions. (**E**) Significantly differential genes associated with the development of the nervous system.

Treatment with 50 μM HCQ resulted in differential expression of 960 transcripts, of which 581 were upregulated and 379 were significantly downregulated as compared to that in the control group (Fig. 5A and Supplementary Table 1).

Gene Ontology (GO) enrichment analysis revealed that expression of genes related to 24 biological processes, 16 cellular components, and 11 molecular functions was significantly modified (Fig. 5D). Among those involved in biological processes, 1322 genes exhibited increased expression and 1489 demonstrated decreased expression. The number of upregulated and downregulated genes related to cellular components was found to be 1062 and 801 respectively. An additional 518 genes were upregulated and 424 were downregulated among those related to molecular functions (Fig. 5D and Supplementary Table 2).

Given the ISH results and abnormal zebrafish development consequent to HCQ treatment, we specifically analyzed the transcriptomics of certain systems. The findings revealed significant differences in 53 genes related to cardiovascular development between the HCQ and control groups, of which 26 genes were downregulated and 27 genes were upregulated (Fig. 5B and Supplementary Table 3).

Additionally, significant differences in the expression of 16 genes related to ocular development were found between the HCQ and control groups, of which five genes were upregulated and 11 genes were downregulated (Fig. 5C and Supplementary Table 4). We found significant differences in 91 genes related to nervous system development in the HCQ group compared with the control group. 46 genes were up-regulated and 45 genes were down-regulated (Fig. 5E and Supplementary Table 4).

**Figure 6:**
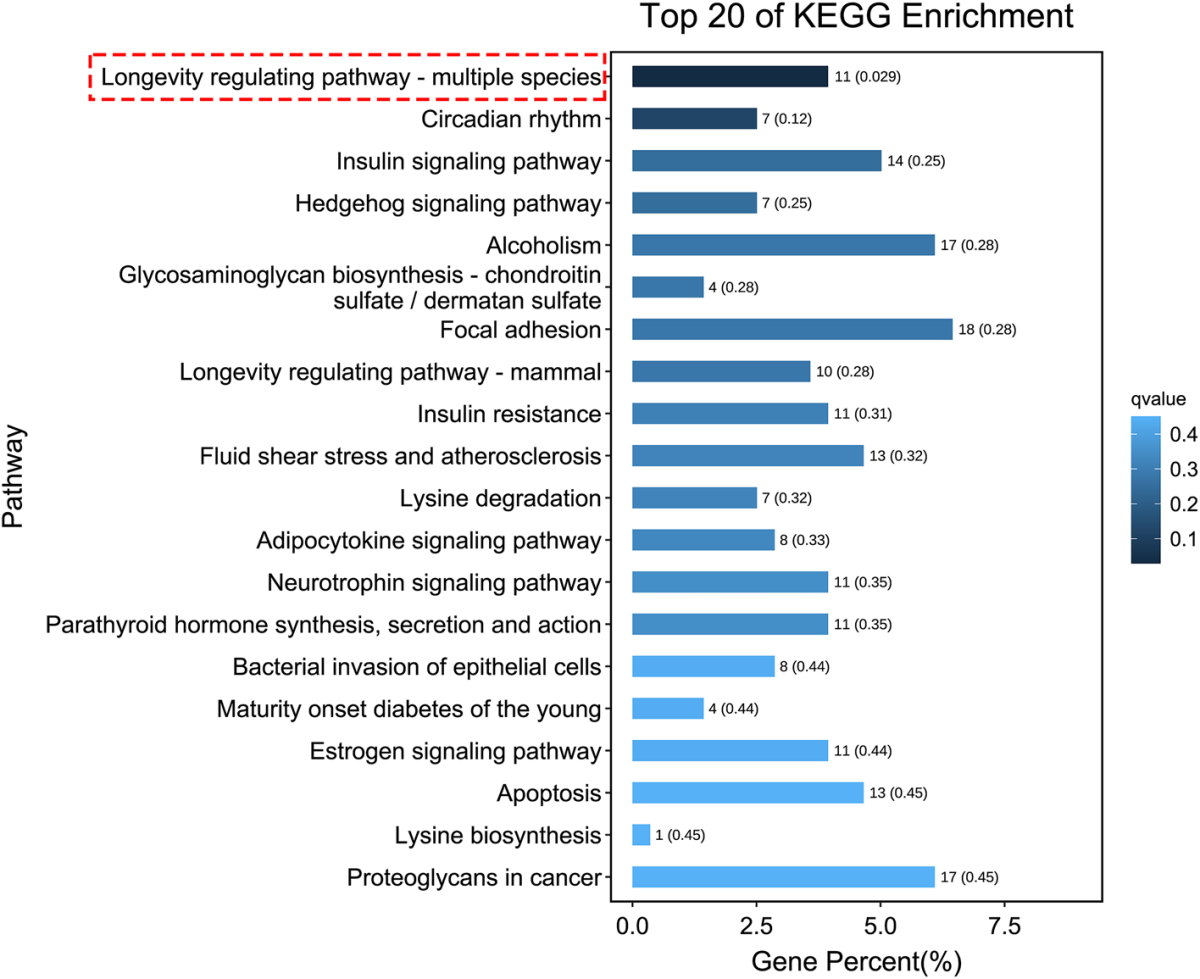
The top 20 enriched KEGG pathways, the pathway selected by the red dashed box is the only one significantly enriched.

To characterize the functions of these differentially expressed transcripts, the Kyoto Encyclopedia of Genes and Genomes (KEGG) pathway-based classification analysis of the ontology of cellular processes, environmental information processing, genetic information processing, human diseases, metabolism, and organismal systems was performed. This revealed that the longevity regulating pathway was the only one which was significantly enriched in the HCQ-treated embryos as compared to that in the control embryos (Fig. 6 and Supplementary Table 5). A previous study demonstrated enhancement of the oxidative stress processes in zebrafish because of HCQ exposure^13^. We found two genes, namely *nfe2* and *pxdn,* that are involved in the oxidative stress process. The gene *nfe2* is also involved in cancer pathways, cardiovascular disease, and aging; however, none of these pathways were found to be significantly enriched (Supplementary Table 6).

## Discussion

Hydroxychloroquine has a good oral absorption, long plasma elimination, and slow renal clearance and crosses the placental barrier, being found at similar concentrations in both the umbilical cord blood and maternal blood^14^. The therapeutic range for SLE was 500–2000 ng/ml (1.15–4.61 μM)**,** and above 2000 ng/ml were considered supratherapeutic^15^. In this study, zebrafish was used as a model organism to study the toxic effect of excessive HCQ on the body. The experimental concentrations were 6.25 μM, 12.5 μM, 25 μM, 50 μM, 100 μM and 200 μM. Is about 5 times, 10 times, 20 times, 40 times, 80 times, 160 times, 320 times of the minimum therapeutic blood concentration, while the starting 6.25 μM is similar to the maximum therapeutic concentration. We observed the phenotype, mortality, hatching rate, body length, heart rate and behavior of zebrafish embryos treated with excessive HCQ, and analyzed the important marker genes such as embryo differentiation and tissue formation by in situ hybridization and transcriptome analysis, in order to obtain the effect of excessive HCQ on the phenotype and function of zebrafish embryos.

This study found that: (1) when the concentration was greater than 50 μM, the embryo mortality increased significantly, the developed abnormalities mortality increased significantly, the hatching rate decreased rapidly; (2) in all of the concentrations of overdose HCQ, the heart rate of zebrafish embryos increased significantly; (3) he movements of larvae in terms of swimming distance and velocity in the HCQ group showed reduced responsiveness to external stimuli as compared to that in the control group. This effect was increasingly apparent with increasing concentrations of HCQ.

In 50 μM HCQ, except the dorsal organizer markers gsc and chd were significantly increased, the ventral marker eve1, the epidermal marker gata2a, the mesoderm marker ntla, the endoderm marker sox17, prox1a and pax2a were no significant change from the control group. but in 100 μM HCQ, all markers were significant change from the control group.

The molecular mechanism of HCQ treatment in terms of its effect on zebrafish embryonic RNA levels was analyzed by using a transcriptome assay. Treatment with 50 μM HCQ resulted in differential expression of 960 transcripts, of which 581 were upregulated and 379 were significantly downregulated as compared to that in the control group Including genes related to ocular development, heart development and nerves.

For the first time, we evaluated the toxicity of overdose HCQ on zebrafish embryos, provided useful information for further research on the toxicity mechanism of HCQ overdose, and provides some insight that can guide future studies in humans.

## Material and methods

### Ethical approval and ethics statement

All experiments in this study were conducted in accordance with ARRIVE guidelines and “Guide for the Care and Use of Laboratory Animals”^16^, and were approved by the Animal Care and Use Committee of West China Second University Hospital, Sichuan University (Approval ID: HXDEYY20131021)^17^.

### Zebrafish lines and embryos

Wildtype (WT) AB strain fish lines were utilized. Staging of the embryos was carried out as previously described^18^. Ages of the embryos are given as hours post-fertilization (hpf) or days post-fertilization (dpf). Zebrafish are reared under a 14 h light/10 h dark cycle and are fed the shrimp larvae 2–3 times a day. The water temperature is maintained at 28 ± 1 °C at pH 7. The day before spawning, two pairs of adult zebrafish are placed in a breeding pond with spawning trays and separate males and females overnight by clapboard. The next morning, the zebrafish mate after removing the clapboard, then collect the embryos and place them in a Petri dish filled with egg-water (60 μg ocean salt/mL) (https://zfin.org/zf_info/zfbook/zfbk.html).

### Reagents

#### HCQ stock solution

HCQ sulfate was purchased from MedChemExpress (MCE) (Cat # HY-B1370, China), for experimental use, a 10 mM stock solution was prepared in ultra-pure water (Millipore Milli-Q^®^ Integral 3 Water Purification System), then storage at – 80 °C (Haier Ultra Low Temperature Freezer DW-86L626).

#### HCQ working solution

Working solutions of HCQ at concentrations of 12.5, 25, 50, 100, 200 μM were prepared by dilution with egg-water [reverse osmosis water containing 60 mg sea salt (Crystal Sea^®^, Aquatic Eco-systems, Inc., Apopka, FL, USA) per liter of water (pH 7.5) with addition of freshly made sodium bicarbonate solution]^19^. All solutions were temporarily stored at 4 °C before use.

### Treatment of zebrafish embryos with HCQ and measurement parameters at each stage

For treatment with HCQ (12.5, 25, 50, 100, 200 μM), 1-cell stage post-fertilization embryos were used to simulate in vivo fertilization. For each experiment leading to the analysis of relevant endpoints, eggs laid at the exact same time were pooled. For each treatment, around 50 embryos (± 5) were placed in each Petri dish containing 20 ml buffered egg water. At 5 hpf, 7 hpf, 24 hpf and 48 hpf stage, we collected embryos and larvae for ISH experiment. We calculated the survival rate at 8 hpf, 1 dpf, 2 dpf and 3 dpf. Hatching rate was calculated after 3 dpf. At 60 hpf, tail coiling activity was videoed. At 80 hpf stage, heart rate was scored. Body length were measured at 5 dpf stage with ImageJ software, iridophore images were acquired at the same stage. Control groups (egg-water) were examined simultaneously.

### Zebrafish embryo in situ hybridization and statistics

Whole-mount in situ hybridization was carried out as previously described in Thisse et al.^20^ and Sun et al.^21^. Antisense probe RNAs for in situ hybridization were synthesized using DIG RNA Labeling Kit (SP6/T7) (Roche) and purified by MEGAclear (Ambion). Grayscale measurement and statistics were performed as previously described^22^. Whole-mount ISH images were taken under same magnification and exposure settings by Nikon SMZ1270 microscopy and standardized them with same software setting. Signal strength gray scale of wholemount ISH images was measured by ImageJ software. Measurement for cardiac looping angle at 2 dpf was done as Peng et al.^23^ and Choudhry and Trede^24^ described^25^. Statistical significance is defined as P < 0.05(*), P < 0.01(**), P < 0.001(***).

### Analysis of the transcriptome

We collected the embryos to RNA extraction and RNA-seq experiments by Genedenovo Biotechnology Co., Ltd (Guangzhou, China) for transcriptomic analysis by RNA-seq as previously described^26^*.* Embryos were collected from control and experiment group treated with 50 μM HCQ at 5 hpf stage.

### Statistical analysis

GraphPad Prism, version 9 (GraphPad Software Inc., CA, USA), was used for statistical analysis. We used the unpaired t test to assess for significant differences in expected frequencies between two groups. The survival analyses were conducted using the log-rank (Mantel–Cox) test. P < 0.05 was considered statistically significant in a two-sided test.

## Supplementary Information

Supplementary Legends.

Supplementary Table 1.

Supplementary Table 2.

Supplementary Table 3.

Supplementary Table 4.

Supplementary Table 5.

Supplementary Table 6.

Supplementary Video 1.

## Data Availability

Raw sequencing data of transcriptome assay from this study has been deposited in the NCBI Sequence Read Achieve (SRA) under the accession number PRJNA879284 (Release date: 2022-09-13, http://www.ncbi.nlm.nih.gov/sra/).
