# Supplementary material for: Toxicology evaluation of overdose hydroxychloroquine on zebrafish (Danio rerio) embryos
Source: Sci Rep. 2022 Oct 29;12:18259. doi: 10.1038/s41598-022-23187-9 (PMC9617536; doi:10.1038/s41598-022-23187-9)
Supplement: Supplementary file 1 — Supplementary Legends. [file 41598_2022_23187_MOESM1_ESM.docx]

supplementary table1 Differentially expressed transcripts of HCQ group in this work.

supplementary table2 Significantly enriched GO entries in this work.

supplementary table3 Genes related to cardiovascular system development in the HCQ group.

supplementary table4 Genes related to eye and optic nerve development in the HCQ group.

supplementary table5 Enriched 27 pathways with P-value < 0.05, of which only one pathway (Longevity regulating pathway) is significantly enriched (Q-value < 0.05).

supplementary table6 Two genes (nfe2 and pxdn) are involved in oxidative stress processes in this work.

supplementary video1 Zebrafish larvae’s responsiveness to external stimuli.

In the first row of the video, the left petri dish is the control group, the middle one is the 12.5μM HCQ group, and the right one is the 25μM HCQ group; in the second row of the video, the left one is the 50μM HCQ group, the middle one is the 100μM HCQ group, and the right one is the 200μM HCQ group.
